# A Midwife’s Diagnosis

**Published:** 1886-01

**Authors:** W. J. Collins

**Affiliations:** The Grove, Texas


### A MIDWIFE'S DIAGNOSIS. (THE DOCTOR NOT NEEDED.)

*By W. J. Collins, M. D., The Grove, Texas.*

For Daniel's Texas Medical Journal.

MRS. J., mother of six children, began labor at six, a. m., the first premonition of which was a "gush of waters" while at the breakfast table. The pains increased in frequency and severity, so I was informed by the old lady who was attendlng her, until three, p. m., same day, at which time I was summoned. On my arrival I was met by the midwife, who said, "everything was all right—head presenting—except the pains did not bear down enough, and that I was not now needed"; but wanted me handy in case all did not go well. Very well; and I was seated as an idle spectator to the parturient efforts. I watched the progress of afiairs about fifteen minutes, when I approached the bedside and asked to be allowed to make an examination, which was reluctantly granted—the old lady still saying all was right. Imagine my feelings when I examined and found a shoulder presentation, a prolapsed and pulseless cord, (second position of the right shoulder) and of course a dead child. I explained matters to the husband, telling him what would have to be done to save the life of the mother, to which he consented. For the past six hours Mrs. J. had suffered most agonizing pains, and was rapidly becoming exhausted. It was evident that prompt action must be taken. So, without assistance, as she lay on the bed, I chloroformed her; introduced the hand, turned and delivered. This was not easily done, for the membranes were ruptured nine hours previously, and the womb held the child as in a vise; and, to increase the difficulty, there was a second child. However, in from five to eight minutes we had succeeded in turning and delivering the first babe—dead as before stated. I now turned my attention to the mother, who, after passing from under chloroform, was well nigh exhausted. I gave her a stimulant which soon revived the flagging pulse.

Another examination was now made which found a breach presentation of the second child. This babe was rapidly delivered by the feet, which I brought down, and was born alive, and is now a fine healthy child three weeks old. The combined weight of both children was seventeen pounds, one a boy weighing nine pounds, the other a girl weighing eight pounds. This is the second shoulder presentation I have had in a practice of fifteen years—occuring with me once in about every six hundred and fifty cases. It is an established fact that mal-positions of the fœtus are more frequent in the case of twins than in a single pregnancy.

The mother did well in every way except the bladder had to be catheterized for three or four days.

I will here remark that I have known more than one precious life lost by the incompetency and ignorance of the attendant, and *I do think legal steps* ought to be taken to prevent these ignoramuses from officiating in the lying-in chambers, thus protecting the health and the lives of our mothers.

